# Intravenous injection of allogenic canine mesenchymal stem cells in 40 client-owned dogs: a safety assessment in veterinary clinical trials

**DOI:** 10.1186/s12917-024-04216-3

**Published:** 2024-08-22

**Authors:** Hee-Seon Cho, Woo-Jin Song, Aryung Nam, Qiang Li, Ju-Hyun An, Jin-Ok Ahn, Hyun-Tae Kim, Su-Min Park, Min-Ok Ryu, Myung-Chul Kim, Ju-Hun Kim, Hwa-Young Youn

**Affiliations:** 1 Laboratory of Veterinary Internal Medicine, Department of Veterinary Clinical Science, College of Veterinary Medicine, Seoul National University, Seoul, 08826 Korea; 2 Laboratory of Veterinary Internal Medicine, Research Institute of Veterinary Science, College of Veterinary Medicine, Jeju National University, Jeju, 63243 Korea; 3 Department of Veterinary Internal Medicine, College of Veterinary Medicine, Konkuk University, Seoul, 05029 Korea; 4 Department of Veterinary Medicine, College of Agriculture, YanBian University, YanJi, JiLin China; 5 College of Veterinary Medicine, Institute of Veterinary Science, Kangwon National University, Chuncheon, 24321 Korea; 6 Department of Clinical Sciences and Advanced Medicine, School of Veterinary Medicine, University of Pennsylvania, Philadelphia, PA USA; 7 BioApplications Inc., Seoul, 04174 Korea

**Keywords:** Mesenchymal stem cells, Cell therapy, Adverse effects, Safety, Dog, Intravenous injection

## Abstract

**Background:**

The aim of this study was to evaluate the adverse effects of allogeneic mesenchymal stem cells (MSCs) transplanted via intravenous infusion in dogs and examine their safety. We performed a retrospective analysis of various clinical assessments, including physical examination, blood tests, and radiographs, and monitored the formation of neoplasms during a 6-month follow-up period in 40 client-owned dogs that received intravenous infusion of adipose tissue-derived MSCs (AT-MSCs) for the treatment of various underlying diseases between 2012 and 2018.

**Results:**

No significant adverse effects of MSC therapy were detected by clinical assessment, blood tests, or radiographic examination in the 6-month follow-up period after the first MSC treatment. Additionally no new neoplasms were observed during this period.

**Conclusions:**

To our knowledge, this study is the first to evaluate the safety aspects (≥ 6 months) associated with intravenous allogeneic AT-MSC infusion. These results suggest that allogenic AT-MSC infusion could be a useful and relatively safe therapeutic approach in canines.

## Background

Mesenchymal stem cell (MSC) therapy has been widely studied for many years because of its therapeutic potential and clinical application in various diseases. The self-renewal capacity of MSCs and their ability to differentiate into cells of various lineages, such as myoblasts, fibroblasts, bones cells, tendons, ligaments, and adipose tissue, make them ideal candidates for applications in regenerative medicine. The MSCs can also regulate excessive immune responses and play a role in regulating the expression of mediators of immune responses; such functions may prove useful in the treatment and regulation of immune-mediated diseases [1]. Another benefit of MSC therapy is convenient isolation and expansion of MSCs.

Various studies have investigated the therapeutic potential and safety of MSC therapy in veterinary medicine. Applications of MSC therapy in dogs have been reported for conditions such as inflammatory bowel disease, pemphigus, refractory atopic dermatitis, chronic osteoarthritis, and intervertebral disk disease [2–6]. The safety and efficacy of MSC therapy have been reported for conditions such as gingivostomatitis, chronic enteropathy, chronic kidney disease, and acute kidney injury in cats [7–11].

However, to the best of our knowledge, no study has investigated the long-term (≥ 6 months) safety aspects of MSC therapy in dogs. Thus, this study aimed to evaluate the adverse effects of MSC transplantation in dogs given an intravenous (IV) infusion of allogeneic MSCs, focusing on safety aspects, based on observations over at least six months. We conducted a retrospective analysis of various clinical assessments, such as physical examination, blood tests, and radiograph results to monitor neoplasm formation during the follow-up period.

## Results

### Characterization of canine adipose tissue-derived MSCs (AT-MSCs)

**Fig. 1:**
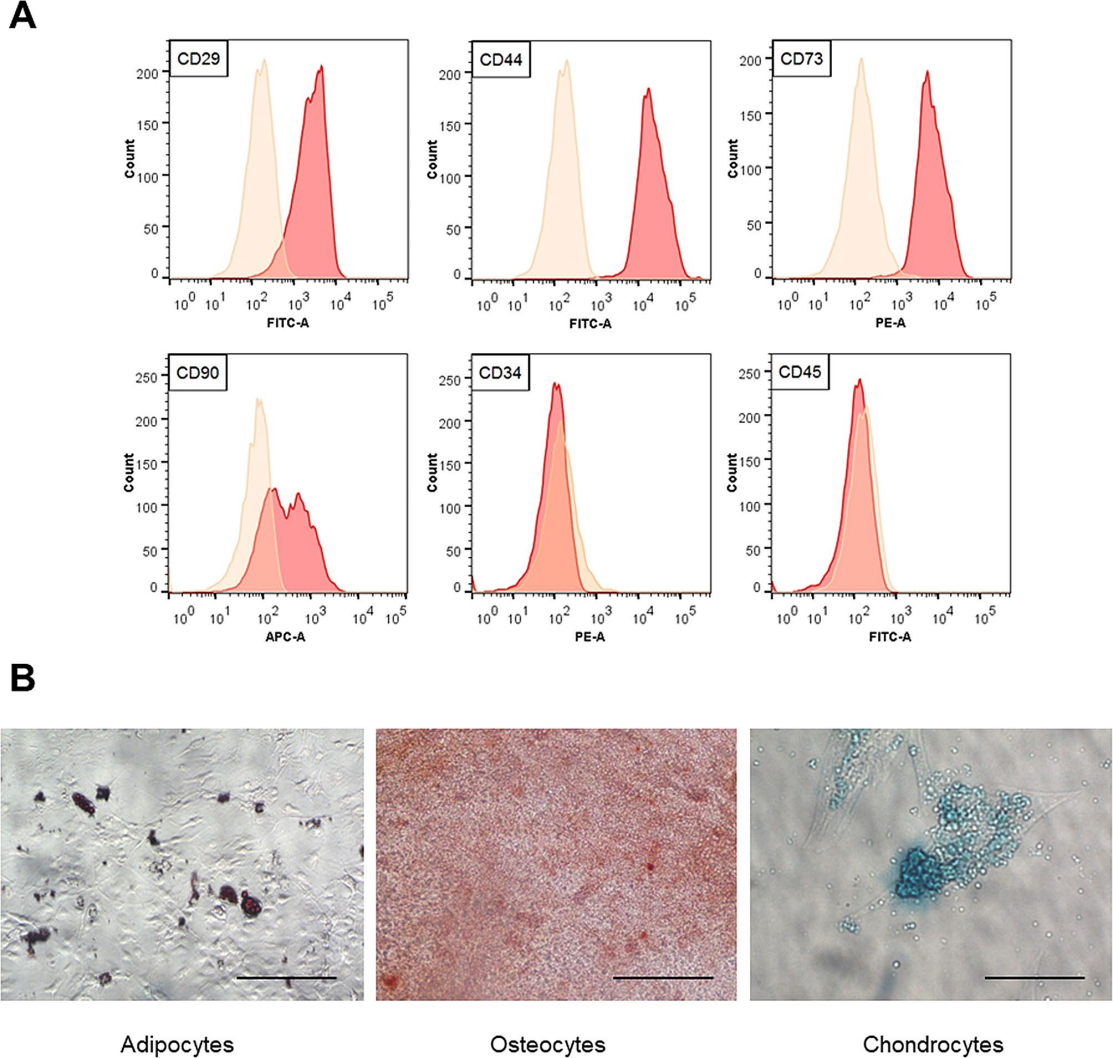
Characterization of mesenchymal stem cells isolated from canine adipose tissue (**A**) Immunophenotype analysis by flow cytometry (**B**) Adipogenic (oil red O staining), osteogenic (alizarin red S staining), and chondrogenic (alcian blue staining) differentiation of canine adipose tissue–derived mesenchymal stem cells. Bars = 200 μm

Cells isolated from canine adipose tissue were characterized by immunophenotyping. Flow cytometry analysis showed that the cells had high expression of stem cell markers, such as cluster of differentiation (CD)29, CD44, CD73, and CD90. However, the cells did not express CD34 or CD45 (Fig. 1A). We also determined the potential of canine AT-MSCs to differentiate into adipocytes, osteocytes, and chondrocytes (Fig. 1B).

### No changes were observed in the various clinical parameters tested before and after MSC therapy

In most dogs, no notable clinical changes were observed between the various parameters assayed before and after MSC treatment (Table 1). Physical examination performed at the hospital revealed no changes in the injection sites. Additionally, there were no changes in systolic blood pressure, pulse rate, respiratory rate, and body temperature following the infusion of allogenic AT-MSCs. Auscultation of cardiac murmurs and lung sounds did not indicate any changes. No swelling, vomiting, or other neurological signs were observed. However, in 3 of the 314 cases (40 dogs), symptoms of pain, flare, and edema were observed in the leg to which the MSC infusion was performed, and the dogs had to visit the hospital again. These patients were diagnosed with vasculitis, and their symptoms improved within a week after appropriate treatment. The complete blood count (CBC) and serum biochemistry profiles showed no specific changes before and after MSC infusion. Adverse effects of MSC treatment, such as pulmonary thromboembolism, pulmonary edema, and hemorrhage, were also not observed in the present study. Additionally, radiographs showed no significant changes before and after MSC infusion. In addition, neoplasms were not identified within the follow-up period (at least 6 months) in any of the cases (Table 1).

**Table 1:** Signalment factors for the dogs enrolled in the study

Patient characteristics	Treated dog
Age (years)	11.2 ± 3.13
Body weight (kg)	6.12 ± 6.55
**Breeds**	
Poodle	5
Maltese	10
Pomeranian	1
Shih tzu	7
Cocker spaniel	3
Yorkshire terrier	3
Mixed	2
Miniature Pinscher	1
White terrier	1
Schnauzers	3
Afghan Hound	2
Pekingese	1
Old English Sheepdog	1
**Sex**	
Castrated male	19
Intact male	2
Intact female	6
Spayed female	13

## Discussion

This study evaluated the adverse effects of MSC transplantation by IV infusion of allogenic MSCs in dogs based on physical examination, blood tests, and radiographs over a period of at least 6 months. Apart from the 3 cases, among the 314 cases (40 dogs) that showed mild vasculitis, no other significant adverse effects of MSC therapy were detected within 6 months of the first MSC treatment. In addition, no new neoplasms were detected within the 6-month follow-up period.

Numerous studies have assessed the safety of intravenously administered MSCs. In a study involving human patients with chronic inflammation, treatment with human umbilical cord blood-derived MSCs (hUBC-MSCs) was reported to be safe and efficient [12]. Another study that performed intravenous administration of allogeneic AT-MSCs in cats with chronic kidney disease reported no significant adverse effects [7, 9]. In two other studies performed on dogs with inflammatory bowel disease and refractory atopic dermatitis, IV infusion of allogenic canine AT-MSCs showed no systemic adverse effects during the follow-up period of six weeks and six months, respectively [4, 6].

However, there are many conflicting reports on the tumorigenic potential of MSCs. Murine bone marrow-derived MSCs undergo malignant transformations in vitro and in vivo [13, 14]. In contrast, another study reported that human MSCs are not susceptible to malignant transformation in long-term in vitro cultures [15]. Furthermore, various studies have reported that the administration of hUCB-MSCs in immunodeficient mice does not result in tumor formation, suggesting that hUCB-MSCs lack tumorigenicity in vivo [16, 17]. However, studies have rarely monitored the long-term risk of tumorigenesis of allogeneic MSC infusion in dogs.

This study has a few limitations. First, the sample size used in this study was small, as only a limited number of dogs satisfied the inclusion criteria. Second, the follow-up period was capped at six months, longer monitoring period is necessary to assess the long-term safety aspects of MSC therapy. Third, due to the nature of retrospective studies, the frequency and intervals of MSC administration varied among individual subjects. Fourth, the evaluation of tumor formation through advanced imaging examinations such as computed tomography, in addition to X-ray or ultrasound, was not performed. Fifth, the observation period of 6 months was not sufficiently long to monitor tumorigenesis. Despite these limitations, observations from this study are important because it is the first study to assess the safety of IV infusion of allogenic AT-MSCs in client-owned dogs with various disorders.

## Conclusion

Intravenous administration of allogenic AT-MSCs in client-owned dogs treated for various diseases did not show any adverse effects, as observed by physical examination, blood tests, and imaging, in a majority of the cases. Thus, allogenic MSC infusion is a useful and a relatively safe therapeutic approach for dogs.

## Methods

### Study population

A total of 40 client-owned dogs that received IV infusion of AT-MSCs for the treatment of various underlying diseases in the veterinary medical teaching hospital between 2012 and 2018 were included in this study. The data are summarized in Table 2. The dogs had a mean age and weight of 11.2 years and 6.12 kg, respectively. This study included patients with a variety of diseases, and the details are summarized in Table 3. The inclusion criteria was the following: (a) patients who received the first IV MSC injection at least six months prior to the start of this study. (b) patients were transplanted more than twice with appropriate follow-up. (c) patients were assessed before transplantation and at each post-treatment follow-up point with history taking, physical examination, blood analysis, thoracic radiography, and abdominal ultrasound.

**Table 2:** Underlying diseases in the dogs enrolled in the study

Disease type	Details (*n*)
Urinary disease	Acute kidney injury [2], Chronic kidney disease [18], Fanconi’s syndrome [1]
Cardiovascular disease	Chronic Valvular Heart Disease [5]
Neurological disease	Hydrocephalus [1], Granulomatous Meningoencephalitis [1]
Hepatobiliary disease	Chronic Hepatitis [3], Liver Cirrhosis [1], Hepatocutaneous syndrome [1]
Gastrointestinal disease	Inflammatory Bowel disease (1), Protein-Losing Enteropathy [1]
Endocrine disease	Diabetes Mellitus [4]
Tumor	Lymphoma [3], Hepatocellular Carcinoma [1]
Immune-mediated disease	Immune-mediated hemolytic anemia [1], Immune-mediated polymyositis [1], Pemphigus foliaceus [1]

**Table 3:** Safety evaluation for intravenous administration of canine allogenic adipose tissue-derived MSCs

Case	Signalment	Underlying disease	Complication after MSC administration	Number of MSC administration	Follow-up period from MSC therapy (Months)	Subsequent tumorigenesis
1	11 year MC Poodle	DM	-	3	14	-
2	12 year FS Maltese	CKD	-	3	16	-
3	13 year FS Pomeranian	DM	-	4	11	-
4	9 year IF Shih-tzu	Chronic hepatitis	-	5	39	-
5	9 year FS Cocker spaniel	CKD	-	2	6	-
6	6 year IF Maltese	Protein losing enteropathy	-	5	25	-
7	13 year MC Yorkshire terrier	CKD, Proteinuria	-	10	21	-
8	6 year MC Cocker spaniel	AKI	-	4	6	-
9	12 year MC Mixed	MMVD	-	8	33	-
10	15 year FS Poodles	DM	-	3	6	-
11	11 year MC Mixed	DM	Vasculitis	5	15	-
12	10 year MC Shih-tzu	MMVD	-	3	35	-
13	13 year MC Maltese	CKD	-	7	20	-
14	9 year FS Poodles	Fanconi syndrome	-	11	29	-
15	16 year FS Miniature pinscher	CKD	-	2	6	-
16	8 year FS Maltese	MMVD	-	4	8	-
17	14 year IF Maltese	Hydrocephalus	-	3	12	-
18	8 year MC White terrier	AKI	-	5	8	-
19	14 year MC Maltese	CKD, Proteinuria	-	6	7	-
20	14 year FS Schnauzer	CKD	-	4	6	-
21	13 year IM Cocker spaniel	CKD	-	2	6	-
22	12 year MC Maltese	Hepatocutaneous syndrome	-	24	18	-
23	7 year IF Afghan hound	Immune mediated polymyositis	-	3	7	-
24	12 year FS Shih-tzu	Hepatocellular Carcinoma	-	51	28	-
25	2 year MC Yorkshire terrier	GME	-	5	6	-
26	12 year MC Shih-tzu	CKD	-	3	6	-
27	13 year MC Schnauzer	Chronic Hepatic Failure	-	4	6	-
28	10 year MC Afghan hound	Multicentric lymphoma	-	16	7	-
29	16 year FS Schnauzer	Chronic Hepatic Failure	-	9	10	-
30	11 year MC Yorkshire terrier	CKD	-	19	20	-
31	15 year FS Maltese	MMVD	Vasculitis	13	16	-
32	7 year FS Poodle	Inflammatory Bowel Disease	Vasculitis	3	14	-
33	13 year MC Pekingese	MMVD	-	2	10	-
34	15 year MC Shih-tzu	Multicentric lymphoma	-	2	7	-
35	11 year IF Poodle	CKD	-	2	24	-
36	11 year FS Old English Sheepdog	Liver cirrhosis	-	9	8	-
37	11 year MC Shih-tzu	Pemphigus foliaceus	-	11	36	-
38	10 year IM Shih-tzu	Alimentary lymphoma	-	27	32	-
39	8 year MC Maltese	Non-regenerative IMHA	-	5	6	-
40	16 year IF Maltese	CKD	-	7	10	-

MC, male castrated; FS, female spayed; IM, intact male; IF, intact female

DM, diabetes mellitus; CKD, chronic kidney disease; AKI, acute kidney injury; MMVD, myxomatous mitral valve disease; GME, granulomatous meningoencephalitis; IMHA, immune-mediated hemolytic anemia; MSC: mesenchymal stem cell

### Isolation and characterization of canine AT-MSCs

Canine adipose tissue was obtained from healthy beagle dogs aged < 2 years old and free of infection by canine distemper virus, parvovirus, and coronavirus. Adipose tissues were obtained using protocols approved by the Institutional Animal Care and Use Committee of Seoul National University (protocol no. SNU-170724-6) performed in accordance with approved guidelines. The MSCs were isolated and characterized as previously described [18]. The tissue samples were briefly washed with phosphate-buffered saline (PAN Biotech, Aidenbach, Germany) containing penicillin (100 U/mL) and streptomycin (100 g/mL) and then cut into small pieces. The cells were then chemically digested at 37 °C using collagenase type IA (1 mg/mL; Sigma-Aldrich, St. Louis, MO, USA) for 1 h. After incubation, Dulbecco’s modified Eagle’s medium (PAN Biotech) containing 10% fetal bovine serum (PAN Biotech) was added to stop enzymatic activity. The tissue was thereafter centrifuged at 1200 × *g* for 5 min and the supernatant was discarded. The pellet was then filtered through a 70-µm Falcon cell strainer (Fisher Scientific, Waltham, MA, USA) and incubated in Dulbecco’s modified Eagle’s medium containing 10% fetal bovine serum at 37 °C in a humidified atmosphere of 5% CO_2_. After 48 h, the cultures were washed five times with phosphate-buffered saline to remove non-adherent cells and centrifuged again. The cells were then incubated with fresh medium, which was changed every 48 h until the cells reached 70–80% confluence, after which they were repeatedly sub-cultured under standard conditions. The cells were also determined to be mycoplasma-free using a Mycoplasma PCR Detection Kit (Cellsafe Co. Ltd., Suwon, Korea).

The cells were characterized by immunophenotyping and multilineage differentiation before use. For immunophenotyping, the isolated cells were evaluated by flow cytometry using fluorescein isothiocyanate (FITC)-, phycoerythrin (PE)-, and allophycocyanin (APC)-conjugated antibodies against the following proteins: CD29-FITC, CD31-FITC, CD73-PE (BD Biosciences, Franklin Lakes, NJ, USA), CD44-FITC, CD45-FITC, and CD90-APC (eBioscience, San Diego, CA, USA). The cells were analyzed using a FACSAria II system (BD Biosciences, city, country). Multilineage differentiation was evaluated using StemPro Adipogenesis Differentiation, StemPro Osteogenesis Differentiation, and StemPro chondrogenesis differentiation kits (all from Gibco/Life Technologies, Carlsbad, CA, USA) according to the manufacturer’s instructions, followed by Oil Red O, Alizarin Red, and Alcian blue staining.

### Administration of canine AT-MSCs

All dogs were administered intravenous chlorpheniramine (0.5 mg/kg) and dexamethasone (0.1 mg/kg) five minutes prior to MSC infusion to prevent hypersensitivity reactions. Immunophenotypic characterization of canine AT-MSCs was performed by flow cytometry, and their differentiation potential was assayed. Allogenic AT-MSCs were administered into the cephalic or saphenous veins through a catheter. Cells were resuspended in 5–10 mL of 0.9% normal saline and 5 × 10^6^ cells/kg were administered by IV infusion performed slowly for 30 min.

### Clinical assessment

Each dog underwent physical examination before and after intravenous allogenic MSC infusion. Physical examination evaluated the following components: blood pressure, body temperature, respiratory rate, pulse rate, auscultation of cardiac murmur and lung sounds, presence of neurological signs, and swelling of the limbs. This study mainly included veterinary patients from the outpatient department, and hence, we conducted history taking from the owners to estimate the changes in appetite or vitality, pain response, edema, or fever during the next visit.

### Blood tests

Data from the CBC and serum biochemistry profiles were assessed to determine adverse reactions to allogenic MSC infusion. Parameters for CBC included red and white blood, platelet, neutrophil, lymphocyte, monocyte, and eosinophil cell counts; packed cell volume; and hemoglobin levels. Additionally, the serum levels of sodium, potassium, chlorine, alkaline phosphatase, alanine aminotransferase, aspartate aminotransferase, gamma glutamyl transferase, total bilirubin, ammonia, total protein, albumin, triglycerides, total cholesterol, glucose, blood urea nitrogen, creatinine, phosphorus, and calcium were also assessed. The CBC and serum biochemical profiles were compared between the values obtained for the parameters before and after MSC treatment. The specific parameters of the blood test varied depending on the underlying disease in the veterinary patients.

### Thoracic radiography

Radiographic examination (EVA-HF525, GEMS, IL, USA) was performed to assess any adverse effects of allogeneic MSC infusion, such as pulmonary edema, hemorrhage, and pulmonary thromboembolism. Right lateral and ventral dorsal thoracic views were obtained for both the inspiratory and expiratory phases. Image analysis was conducted by veterinarians of the relevant department. The radiographs were then assessed and compared to evaluate whether there was a significant change in the thoracic radiograph before and after MSC injection.

## Data Availability

The datasets used and analyzed during the current study are available from the corresponding author on reasonable request.

## Abbreviations

MSCs: Mesenchymal stem cells
AT-MSCs: Adipose tissue-derived MSCs
IV: Intravenous
CD: Cluster of differentiation
CBC: Complete blood count
hUBC-MSCs: Human umbilical cord blood-derived MSCs
FITC: Fluorescein isothiocyanate
PE: Phycoerythrin
APC: Allophycocyanin
